# Radiation Dose Escalation Is Crucial in Anti-CTLA-4 Antibody Therapy to Enhance Local and Distant Antitumor Effect in Murine Osteosarcoma

**DOI:** 10.3390/cancers12061546

**Published:** 2020-06-12

**Authors:** Wataru Takenaka, Yutaka Takahashi, Keisuke Tamari, Kazumasa Minami, Shohei Katsuki, Yuji Seo, Fumiaki Isohashi, Masahiko Koizumi, Kazuhiko Ogawa

**Affiliations:** 1 Department of Medical Physics and Engineering, Osaka University Graduate School of Medicine, Suita, Osaka 565-0871, Japan; takewata216@gmail.com (W.T.); skatsuki@sahs.med.osaka-u.ac.jp (S.K.); koizumi@sahs.med.osaka-u.ac.jp (M.K.); 2 Department of Radiation Oncology, Osaka University Graduate School of Medicine, Suita, Osaka 565-0871, Japan; tamari@radonc.med.osaka-u.ac.jp (K.T.); k_minami@radonc.med.osaka-u.ac.jp (K.M.); seo@radonc.med.osaka-u.ac.jp (Y.S.); isohashi@radonc.med.osaka-u.ac.jp (F.I.); kogawa@radonc.med.osaka-u.ac.jp (K.O.)

**Keywords:** radiation, immune checkpoint blockade, abscopal effect, dose escalation, osteosarcoma

## Abstract

We previously reported that a combination of 10 Gy of X-ray irradiation and dual immune checkpoint blockade with anti-CTLA-4 (C4) and anti-PD-L1 antibodies produced a significant shrinkage of irradiated and unirradiated tumors (abscopal effect) and prolonged overall survival. However, the optimal radiation delivery regimen combined with single immune checkpoint blockade of C4 for inducing a maximum systemic antitumor response still remains unclear, particularly for patients with osteosarcoma. We used syngeneic C3H mice that were subcutaneously injected with LM8 osteosarcoma cells into both legs. C4 was administered three times, and one side of the tumor was irradiated by X-ray beams. The optimal radiation dose required to induce the abscopal effect was explored with a focus on the induction of the type-I interferon pathway. Radiation delivered in a single fraction of 10 Gy, 4.5 Gy × 3 fractions (fx), and 2 Gy × 8 fx with C4 failed to produce significant inhibition of unirradiated tumor growth compared with monotherapy with C4. Dose escalation to 16 Gy in a single fraction, or the equivalent hypofractionated dose of 8 Gy × 3 fx, which significantly increased secretion of IFN-β in vitro, produced a dramatic regression of both irradiated and unirradiated tumors and prolonged overall survival in combination with C4. Furthermore, irradiation at 16 Gy in both a single fraction and 8 Gy × 3 fx diminished regulatory T cells in the unirradiated tumor microenvironment. These results suggest that total dose escalation of radiation is crucial in C4 therapy to enhance the antitumor response in both local and distant tumors and prolonged overall survival regardless of fractionation for osteosarcoma.

## 1. Introduction

Radiotherapy (RT) has been a component of the standard treatment against many types of solid tumors and is highly effective for local control. It has been widely reported that tumor regression is observed, even in distant tumors out of the radiation field, when RT is utilized for patients with multiple metastasis [1,2,3,4]. This phenomenon is an immune-mediated event known as the abscopal effect [5,6,7,8,9,10]. However, the abscopal effect is rarely observed in treatment of cancers by RT alone, both in preclinical models and in clinical practice. Recently, Honjo et al. and Allison et al. discovered PD-1 and CTLA-4 antibodies, respectively, which have revolutionized treatments for patients with multiple metastases in various types of cancers, providing a durable long-term response [11,12,13,14,15,16]. However, the response is observed for only up to 30% of patients [11,12,13,14,15,16]. Together with the radiation-induced abscopal effect, combined therapy with immune checkpoint blockade has been found to be an effective strategy for some cancers [17,18,19,20,21,22,23], although the optimal combination strategy, especially in the context of radiation delivery (e.g., sequence, total dose, and fractionation) remains unclear for some types of tumors.

Osteosarcoma is one of the most common cancers in adolescence and young adults and often leads to lethal pulmonary metastasis [24,25,26]. The current standard treatment for osteosarcoma is a combination of radical surgery and chemotherapy such as doxorubicin and methotrexate [26,27,28]; however, the efficacy is still limited, indicating that developing a novel therapeutic strategy is an emergent issue [29,30]. Recently, our group reported that dual immune checkpoint blockade of anti-PD-L1 (P1) and anti-CTLA-4 (C4) antibodies combined with single delivery of 10 Gy X-ray irradiation enhanced the systemic antitumor response both at local and distant sites in murine osteosarcoma [31]. Although this triple combination therapy produced a fascinating treatment outcome without significant adverse effect in the preclinical model, treatment using dual immune checkpoint inhibitors significantly increases the risk of immune-related adverse events for patients [32,33,34]. Moreover, considering the medical expense, exploring novel therapeutic strategies with single immune checkpoint blockade without attenuating treatment efficacy is an urgent issue. Although the addition of radiation to single immune checkpoint blockade therapy is expected to bring an enhanced antitumor effect to some extent, the optimal radiation delivery regimen, including total radiation dose and fractionation, required to exert a greater systemic response remains unclear, particularly for osteosarcoma. On the basis of the recent evidence that the induction of type-I interferons is associated with radiation-induced antitumor immunity [35], we show that a high, but not intermediate, dose of local irradiation is necessary to exert the maximum efficacy of the anti-CTLA-4 antibody (C4) for osteosarcoma.

## 2. Results

### 2.1. C4 Monotherapy Transiently Inhibits Tumor Growth

**Figure 1:**
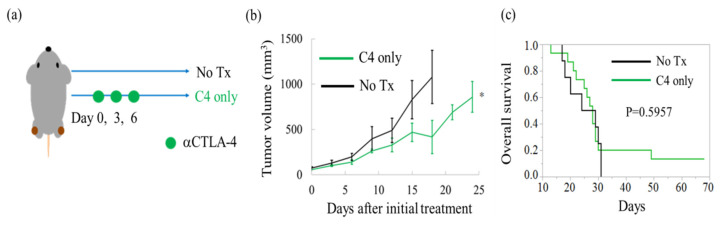
Efficacy of anti-CTLA-4 antibody (C4) for the treatment of murine osteosarcoma. (**a**) Schema of the treatment schedule. Treatment was initiated from the date defined as day 0 on which tumor volume reached *≥*14 mm^3^. C4 was administered three times every 3 days. (**b**) Tumor volume change from the initial treatment. (**c**) Overall survival by the Kaplan–Meier method. Error bars represents SEM. * *p* < 0.05.

We first investigated whether monotherapy of C4 was effective for suppressing osteosarcoma in an LM8 tumor-bearing mouse model (Figure 1a). Significant but transient regression was observed on day 18 (*p* = 0.0478) of C4 monotherapy (Figure 1b). However, the overall survival of the C4 group was not significantly prolonged compared with that of the No Tx group (*p* = 0.5957), indicating that three cycles of C4 monotherapy contribute to tumor growth delay to some extent but not to prolonged overall survival (Figure 1c).

### 2.2. Intermediate Radiation Dose with Concurrent C4 Therapy Does Not Adequately Enhance the Antitumor Efficacy

**Figure 2:**
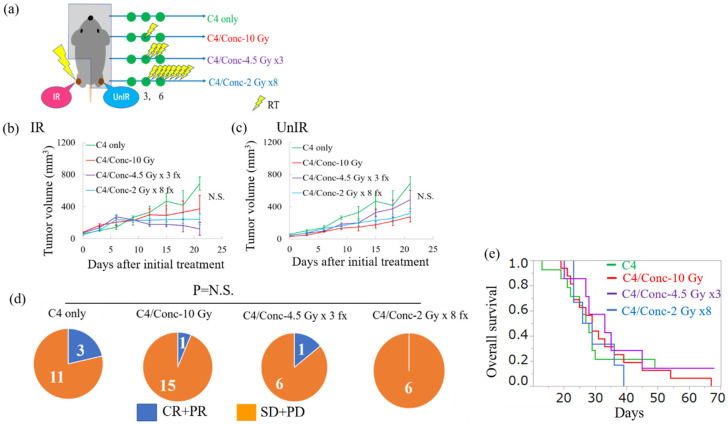
Antitumor efficacy of C4 therapy with or without X-ray at 10 Gy or its equivalent dose in normal fraction or hypofraction. (**a**) Schema of the treatment schedule with mouse setup to irradiate one side of the tumor, with the other protected using *≥*4 mm-Pb lead plates. Treatment was initiated on day 0 when tumors reached a size of 14 mm^3^. C4 was administered on days 0, 3, and 6. (**b**,**c**) Tumor volume change after initial treatment of IR and UnIR tumors. (**d**) Pie charts showing the ratio between complete and partial response (CR + PR; the longest diameter on day 21 ≤ that on the day of the initial treatment) and stable and progressive disease (SD + PD) of the abscopal tumor. (**e**) Overall survival by the Kaplan–Meier method. Error bars represent SEM. The data of C4 only are shared with Figure 1 because the materials and procedures were exactly the same.

We previously demonstrated that the concurrent triple combination of P1, C4, and 10 Gy of X-ray irradiation enhanced the antitumor efficacy for both local and distant tumors [31]. Therefore, we investigated whether single immune checkpoint blockade of C4 with concurrent X-ray irradiation at 10 Gy, and its equivalent doses in fractionation determined by a linear–quadratic model [36,37], 4.5 Gy × 3 fx, and 2 Gy × 8 fx, still enhanced antitumor efficacy (Figure 2a). Despite the direct irradiation at the above dose levels to the tumor, only a slight delay in tumor growth was observed in the irradiated (IR) tumor compared with the C4 only group (Figure 2b). No significant difference was observed in the volume of the unirradiated (UnIR) tumor on the other side of the leg (Figure 2c). To analyze the likelihood of the abscopal effect, we examined the ratio of complete and partial response (CR + PR), defined as the longest diameter on day 21 that was shorter than that on the day of the initial treatment. Response was observed in 3 of 14 mice in the C4 only group, and addition of 10 Gy, 4.5 Gy × 3 fx, or 2 Gy × 8 fx to C4 did not increase the response. Specifically, 1 of 15 in the C4/Conc-10 Gy group, 1 of 7 in the C4/Conc-4.5 Gy × 3 fx group, and 0 of 6 in the C4/Conc-2 Gy × 8 fx group exhibited CR + PR in the abscopal site (Figure 2d). No statistical significance was observed among these groups. Furthermore, the comparison of the responder in the abscopal tumor between the C4 monotherapy and C4 with the intermediate-dose regimen (10 Gy, 4.5 Gy × 3 fx, and 2 Gy × 8 fx) revealed no significant differences (Figure S1). The long-term follow-up revealed that no significant survival benefit was observed in either combination therapy compared with C4 only. The median survival times of C4 only, C4/Conc-10 Gy, C4/Conc-4.5 Gy × 3 fx, and C4/Conc-2 Gy × 8 fx were 27.5, 29, 33, and 27.5 days, respectively (Figure 2e).

### 2.3. Dose Escalation Dramatically Induces the Type-I Interferon Pathway In Vitro

**Figure 3:**
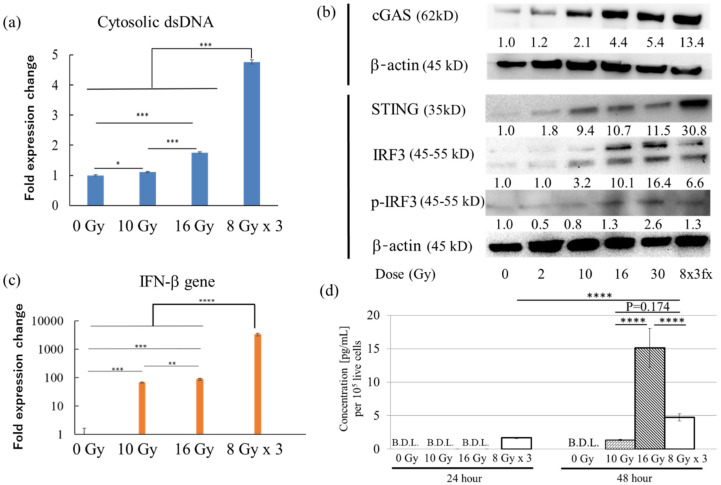
Analysis of the type-I interferon pathway in vitro. (**a**) Fold expression change in cytosolic double-stranded DNA expression 24 h after the last delivery of irradiation. (**b**) Expression level of type-I interferon-related proteins by Western blotting 24 h after the last delivery of irradiation. Values on each band represent the area density of the proteins normalized to a loading control (β-actin). Untrimmed pictures in the Western blot are provided in Supplemental Figure S3. (**c**) Fold expression change of *Ifnb1* coding IFN-β 24 h after the last delivery of irradiation. (**d**) Concentration of IFN- β in the cell culture supernatant 24 and 48 h after the final delivery of irradiation at 0 Gy, 10 Gy, 16 Gy, or 8 Gy × 3fx. Error bars represent SD. * *p* < 0.05 comparing each radiation dose. ** *p* < 0.01, *** *p* < 0.001, **** *p* < 0.0001. Abbreviation: B.D.L.: bellow detection limit.

Since the C4 treatment with a single fraction of 10 Gy and the equivalent fractionated doses showed no significant reduction in the IR and UnIR tumors or extension of overall survival compared with the C4 only group, we next examined whether further dose escalation increased the antitumor immunity. A recent study demonstrated that high-dose hypofractionated irradiation at 8 Gy × 3 fx significantly induced immune response through type-I interferon pathways both in vitro and in vivo [35]. Accumulation of the cytosolic double-stranded DNA (dsDNA) induced by DNA damage triggers IFN-β production through the cyclic GMP-AMP synthase (cGAS) stimulator of the interferon gene (STING) pathway [38,39,40,41]. Our in vitro results showed that irradiation at 10 Gy in a single fraction significantly increased the cytoplasmic dsDNA level compared with 0 Gy, but a higher expression level was observed at 16 Gy in a single fraction. Notably, 8 Gy × 3 fx provided the highest dsDNA level (Figure 3a). The expression of cGAS, a sensor protein of cytosolic DNA, was upregulated in a dose-dependent manner. Notably, 8 Gy × 3 fx dramatically increased the expression level. STING, which is activated by cGAS, is strongly expressed in a dose-dependent manner. Importantly, its downstream signal, phosphorylation of interferon regulatory factor 3 (phospho-IRF3), is expressed to a greater extent by 16, 30 Gy, and 8 Gy × 3 fx irradiation than 0 Gy but not by 2 Gy and 10 Gy irradiation (Figure 3b, Figure S2).

We next examined the change in expression of Ifnb1 coding IFN-β. Ifnb1 was increased in a dose-dependent manner by 68-fold, 88-fold, and 3304-fold at 10, 16 Gy, and 8 Gy × 3 fx compared with that at the 0 Gy sample, respectively (Figure 3c). Secretion of IFN-β into the cell culture supernatant was also increased by 8 Gy × 3 fx but was not detected by 10 or 16 Gy irradiation 24 h after the final delivery of irradiation (Figure 3d). However, the increase was more notable at 16 Gy than 8 Gy × 3 fx and 10 Gy 48 h after irradiation (Figure 3d), whereas no increase was observed 24 h after irradiation at a low dose of 2 Gy nor at an extremely high dose of 30 Gy. However, 30 Gy irradiation exhibited significantly increased secretion 48 h after the irradiation, whereas 2 Gy did not (Figure S2). Due to the possible normal tissue toxicity at 30 Gy irradiation in a single delivery in vivo, we used 8 Gy × 3 fx and 16 Gy for further in vivo experiments.

### 2.4. Concurrent Combination of Anti-CTLA-4 and High-Dose Irradiation Dramatically Enhanced Antitumor Efficacy at Both Local and Distant Sites

**Figure 4:**
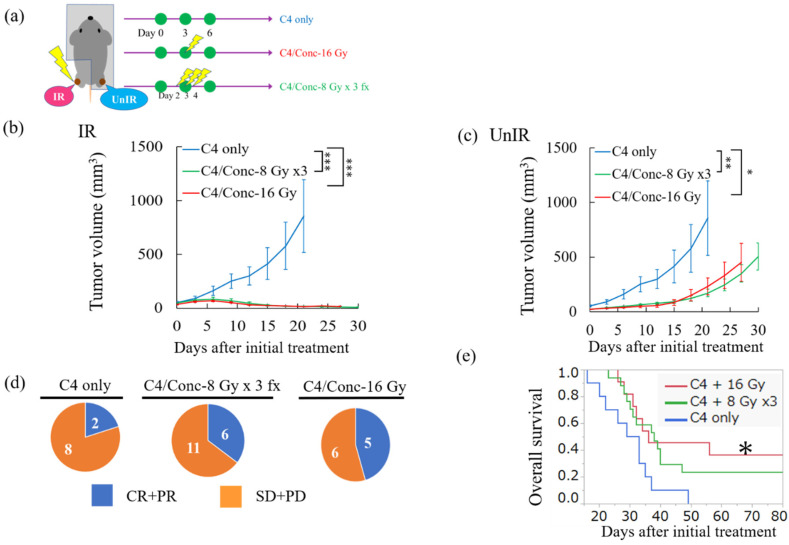
Antitumor efficacy of C4 therapy with or without X-ray at 16 Gy or its equivalent dose in hypofraction. (**a**) Scheme of the treatment schedule. Day 0 was defined as the day when tumors reached a size of 14 mm^3^. (**b**) Tumor volume change at the irradiated side and (**c**) unirradiated side. (**d**) Pie charts showing the ratio between complete and partial response (CR + PR; the longest diameter on day 21 ≤ that on the day of the initial treatment) and stable and progressive disease (SD + PD) of the abscopal tumor. (**e**) Overall survival using the Kaplan–Meier method. Error bars represent SEM. * *p* < 0.05, ** *p* < 0.01, *** *p* < 0.001.

We next investigated whether a higher radiation dose provided a robust antitumor effect both at the in-field and abscopal lesion in vivo (Figure 4a). IR tumors were eradicated in 14 of 17 mice in the C4/Conc-8 Gy × 3 fx group and in 9 of 11 mice in the C4/Conc-16 Gy group, which was a significantly higher response than the C4 only group (Figure 4b).

Interestingly, the UnIR tumor volume was also significantly decreased in both the C4/Conc-8 Gy × 3 fx and C4/Conc-16 Gy groups (Figure 4c). Only 2 in 10 mice in the C4 only group responded in the abscopal tumors, whereas 6 in 17 mice and 5 in 11 mice responded in the C4/Conc-8 Gy × 3 fx and C4/Conc-16 Gy groups, respectively (Figure 4d). Moreover, the overall survival was significantly prolonged in the 8 Gy × 3 fx and 16 Gy combined with C4 therapy groups compared with the C4 only group. The median survival times of the C4 only, C4/Conc-8 Gy × 3 fx, and C4/Conc-16 Gy groups were 31, 38, and 36 days, respectively (Figure 4e). Sequential combination therapy with irradiation at 8 Gy × 3 fx prior to C4 administration (C4/Pre-8 Gy × 3 fx) exhibited comparable antitumor efficacy in both the IR and UnIR tumors (Figure S4a–c).

**Figure 5:**
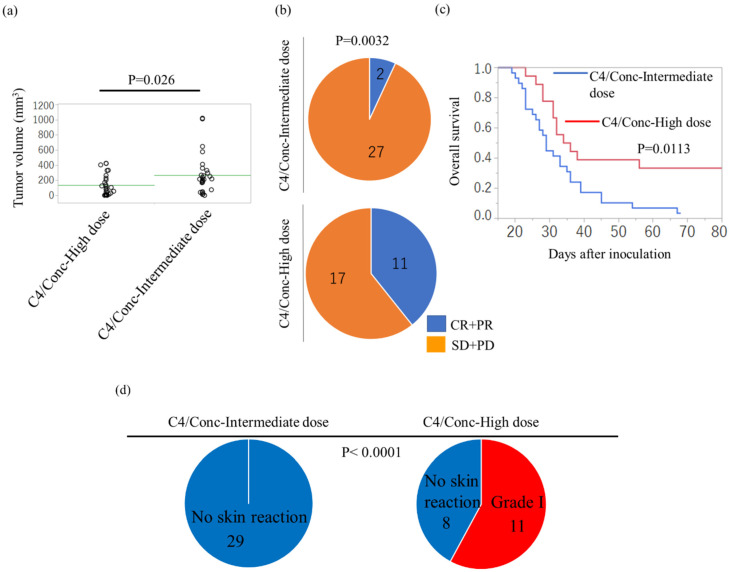
Comparison of the treatment efficacy in the abscopal tumor and overall survival between intermediate-dose (10 Gy, 4.5 Gy × 3 fx, and 2 Gy × 8 fx) and high-dose (16 Gy, 8 Gy × 3 fx) regimens. (**a**) Comparison of the treatment efficacy in abscopal tumor volume. (**b**) Pie charts showing the ratio between complete and partial response (CR + PR; the longest diameter on day 21 ≤ that on the day of the initial treatment) and stable and progressive disease (SD + PD) of the abscopal tumor. (**c**) Overall survival by the Kaplan–Meier method. (**d**) Pie charts showing the ratio between Grade I acute skin reaction and no acute reaction of the irradiated tumors. The data of Figure 2 and Figure 4 were compared because the materials and procedures were exactly the same. Error bars represent SEM.

The direct comparison of the abscopal effect between the intermediate-dose regimen (10 Gy, 2 Gy × 8 fx, and 4.5 Gy × fraction) and the high-dose regimen (16 Gy and 8 Gy × 3 fx) with C4 revealed a significant tumor volume reduction following treatment with the high-dose regimen (Figure 5a). Furthermore, the proportion of the responders at the abscopal sites in the intermediate regimen was 2/29, whereas that in the high-dose regimen was 11/28 (Figure 5b); based on this, the overall survival was significantly prolonged (Figure 5c).

Radiation-induced skin reaction based on the Common Terminology Criteria for Adverse Events v3.0. [42] revealed that Grade 1, which is defined as skin changes such as dry desquamation with generalized erythema, was not observed in mice with the C4/Conc-intermediate dose, whereas 11/19 mice experienced a Grade I skin reaction (Figure 5d). However, the skin reaction recovered within nine days. No radiation-induced systemic symptoms, including apparent slimming and prostration after treatments, were observed in mice in both treatment regimens.

### 2.5. Radiation Dose Escalation Alters the Immune Microenvironment

**Figure 6:**
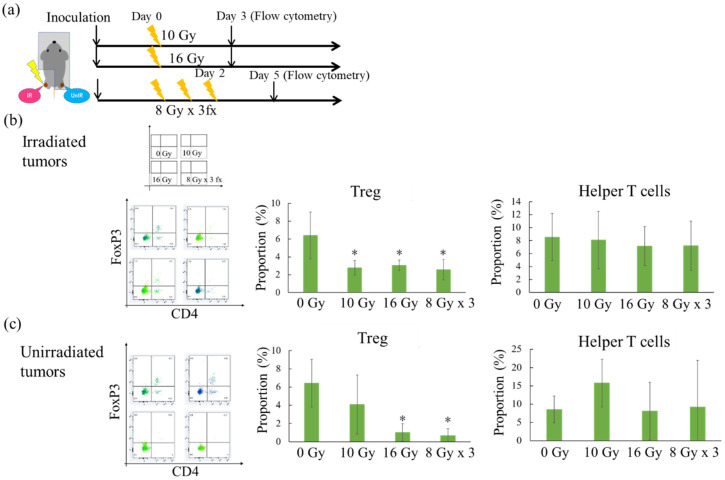
Alteration of the immune microenvironment. (**a**) Schematic diagram for flow cytometry. (**b**) Representative dot plots of CD4 and FoxP3. The middle and right graphs represent the quantifications of CD4 + FoxP3 + Treg and CD4 + FoxP3 − helper T cells in the irradiated tumors, respectively. (**c**) The corresponding data of the unirradiated tumors. * *p* < 0.05.

Since higher dose irradiation enhanced the antitumor efficacy of C4 and prolonged overall survival, flow cytometric analysis was performed to investigate the immune microenvironment in IR and UnIR tumors (Figure 6a). Although regulatory T cells (Tregs) play a crucial role in immune tolerance [43], in the tumor microenvironment, Tregs negatively contribute to the antitumor immune response [43]. Our flow cytometric analysis showed that CD4 + Foxp3 + Treg infiltration in IR tumors was significantly diminished with a single-fraction X-ray of 10 Gy, 16 Gy, and 8 Gy × 3 fx (Figure 6b). Interestingly, Tregs were significantly reduced in UnIR tumors by 16 Gy and 8 Gy × 3 fx irradiation but not with 10 Gy irradiation (Figure 6c).

## 3. Discussion

Osteosarcoma has long been considered as X-ray- and immunotherapy-resistant [26,44]. However, our former study revealed that dual immune checkpoint blockade of P1 and C4 and concurrent use of 10 Gy X-ray irradiation successfully enhanced systemic antitumor immunity, which lead to a significant abscopal effect and prolonged survival in a murine osteosarcoma model [31]. However, the therapeutic response of osteosarcoma to single immune checkpoint blockade combined with various radiation delivery regimens remained unclear. To the best of our knowledge, this is the first evidence that revealed the necessity of radiation dose escalation regardless of the number of fractionations to enhance systemic antitumor efficacy, especially in combination with C4 for osteosarcoma. Although three–four cycles of anti-CTLA-4 antibody are administered in clinical trials of sarcoma and other tumor patients [45,46,47], we administered three cycles of anti-CTLA-4 antibody in the present study based on previous preclinical reports [22,35,47], allowing us to compare our data with other reports. Under this preclinical setting, we found that radiation dose escalation was crucial to induce sufficient systemic anticancer efficacy, even with the use of single checkpoint blockade of C4, and regardless of single-fraction or hypofraction delivery. However, an intermediate radiation dose (i.e., 10 Gy, 4.5 Gy × 3 fx, and 2 Gy × 8 fx) failed to enhance the antitumor efficacy in C4 therapy. Recently, Dewan MZ et al. and Vanpouille-Box et al. demonstrated that high-dose hypofractionated RT, but not the single delivery of 8 Gy nor 20 Gy, evoked a sufficient systemic antitumor effect in combination with C4 in murine mammary and colon carcinoma models [22,35]. Similar to their findings, our results confirmed that the single delivery of 10 Gy irradiation did not enhance antitumor efficacy both in IR and UnIR tumors, resulting in comparable survival with monotherapy of C4.

To find the optimal radiation dose to induce the type-I interferon pathway, we first conducted in vitro experiments prior to conducting in vivo experiments based on a previous report [35]. The type-I interferon pathway plays an important role in adaptive immunity to external stimuli such as viral infections and radiation [48,49]. Cytoplasmic dsDNA activates the cGAS/STING/IRF3 pathway leading to secretion of type-I interferons such as IFN-β [38,50]. Increasing evidence has suggested that dsDNA accumulates in the cytosol after irradiation [35,51,52,53,54]. Indeed, Vanpouille-Box et al. demonstrated that accumulation of dsDNA, upregulation of Ifnb1, and release of IFN-β were only induced following high-dose hypofractionated irradiation but not low-dose nor high-dose irradiation in a single fraction [35]. In contrast, our data revealed that cytoplasmic dsDNA accumulation dramatically increased not only at hypofractionation (i.e., 8 Gy × 3 fx) but also at high dose (i.e., 16 Gy). Accordingly, the expression of its downstream signal, phospho-IRF3, which is one of the transcription factors of Ifnb1, was activated by a high dose in either a single fraction or hypofractionation. Moreover, our gene expression analysis on Ifnb1 also showed a striking correlation with the change in dsDNA expression. However, secretion of IFN-β did not correlate with the accumulation of dsDNA or IFN-β gene expression. Similar to the finding by Vanpouille-Box et al. [35], our data also revealed that 8 Gy × 3 fx significantly promoted the release of IFN-β 24 h after irradiation, whereas 10 Gy did not. Unlike the report of Vanpouille-Box et al, our data revealed that high-dose delivery in a single fraction still induced IFN-β secretion at a later time point than in hypofractionated irradiation. Moreover, the amount of secreted IFN-β was even greater at a high dose (i.e., 16 Gy) and extremely high dose (i.e., 30 Gy) in a single fraction 48 h after irradiation. These results suggest that high-dose irradiation is crucial for triggering the immune response, regardless of fractionation for osteosarcoma. In support of this, our data revealed that both 8 Gy × 3 fx and 16 Gy of irradiation provided equally robust tumor growth delay in both IR and UnIR sites in combination with C4 in vivo. Consistently with this finding, the overall survival was significantly extended, again suggesting that not only hypofractionation but also a single fraction with a high dose are necessary to induce local and systemic control.

The LM8 cells/C3H mice model easily induce lung and liver metastasis [31,55,56]. Our study showed that the majority of deaths in treated or untreated mice were due to distant metastasis, which was confirmed by necropsy, clearly indicating that overall survival reflects the likelihood of distant metastasis. Thus, tumor growth delay in the abscopal tumor with the prolonged overall survival in the C4 with high-dose radiation group reflects the distant metastasis inhibition to a large extent.

Furthermore, we found that a combination of C4 with high-dose RT did not induce severe systemic adverse events, including apparent body weight loss, apparent slimming, and prostration, after the treatments except for those mice just before death due to metastatic burden. In contrast, the high-dose RT in our study induced a Grade I local skin reaction based on the criteria of radiation-induced skin reaction [42]. However, the skin reaction recovered within nine days, suggesting that C4 with high-dose radiation is a tolerable treatment option in this model.

To understand how the optimal dose of irradiation affects the immune cell distribution in IR and UnIR tumors, we further investigated the components of the tumor microenvironment. Tregs were downregulated in both IR and UnIR lesions by high-dose irradiation at 8 Gy × 3 fx and 16 Gy. In contrast, 10 Gy irradiation decreased Tregs only in IR tumors, but only a slight decrease was observed in UnIR tumors at this dose level. Previous reports demonstrated that naïve T cells differentiate into type-17 helper T cells when TGF-β and IL-6 coexist but differentiate into Tregs in the presence of TGF-β and absence of IL-6 [57]. Yagi et al. reported that IL-6 secretion was increased by 16 Gy in a single fraction (delivered to both the hind limbs of mice) [58]; hence, Tregs may be reduced after high-dose radiation not only in IR tumors but also in UnIR tumors. Taken together, regardless of fractionation, high-dose radiation, but not low and intermediate doses, facilitates a favorable immune microenvironment. Thus, irradiation at 8 Gy × 3 fx and 16 Gy combined with C4 could enhance the systemic efficacy of C4, leading to sufficient suppression of both IR and UnIR tumors and extension of survival in mice with osteosarcoma.

Overall, our data revealed that high-dose local irradiation can almost eradicate local tumors and is a crucial factor in osteosarcoma in mice. Further dose escalation may provide better local control and freedom from distant metastasis. However, even the use of high-precision radiation therapy techniques limits the dose escalation because of possible normal tissue toxicities. Recently, our group reported that carbon ion irradiation with dual immune checkpoint blockade enhanced the antitumor effect in both local and distant sites in vivo [56]. Carbon ion beams have a superior dose distribution that can achieve a highly focused dose to targets with rapid dose fall-off nearby normal tissues [59]. Therefore, dose escalation using carbon ion beams with C4 may provide a better antitumor immune response with more decreased toxicity than photon beams in carbon ion irradiation. Further studies are necessary to achieve much better control of local and distant sites for osteosarcoma. In addition, sarcoma inoculated into bone reflects the effect of the microenvironment on the immune response, including the type-I interferon pathway, which is a more clinically relevant approach. Further studies are necessary to investigate the efficacy and underlying mechanisms of C4 with radiation for sarcoma under bone microenvironments.

## 4. Materials and Methods

### 4.1. Ethics Statement

All animal experiments were approved by Osaka University Institutional Animal Care and Use Committee on 2/14/2018 (Approve number; 30-014-005) and performed using the minimum-distress procedure. Mice were basically observed daily and were humanely sacrificed with CO_2_ or sevoflurane inhalation when they met the following humane endpoints: dysfunction of walking, rotating, and longest tumor diameter exceeding 20 mm.

### 4.2. Cells and Reagents

The highly metastatic murine osteosarcoma cell line LM8 was purchased from RIKEN (Saitama, Japan). Cells were cultured in Dulbecco’s modified Eagle’s medium containing 10% fetal bovine serum, 1% penicillin/streptomycin, and L-glutamine, at 37 °C in an incubator at 5% CO_2_ atmosphere.

### 4.3. In Vivo Experiment

Six to seven-week-old C3H mice were purchased from Nihon-Clea (Tokyo, Japan) and were maintained under specific pathogen-free conditions in Osaka University. The mice were subcutaneously inoculated with 3 × 10^5^ LM8 cells into the hind of both thighs (Figure 1a). To evaluate the efficacy of monotherapy with the anti-CTLA-4 antibody (clone: 9H10, Bio × Cell, Lebanon, NH, USA), mice were assigned to No TX (received no treatment) or C4 only (150 µg × 3 times of C4 administration) groups as shown in Figure 1b. To evaluate the efficacy of combination therapy of C4 with concurrent use of X-ray at 10 Gy or its equivalent dose in fractionation, mice were randomly assigned to the following four groups: C4 only, C4/Conc-10 Gy (C4 and concurrent use of X-ray irradiation at 10 Gy), C4/Conc-4.5 Gy × 3 fx (combinatorial therapy of C4 and hypofractionated radiation), or C4/Conc-2 Gy × 8 fx (clinically daily fractionated RT with C4 administration) (Figure 2a). To investigate the benefit of dose escalation for the local and abscopal effect, mice were randomly assigned to three groups as follows: C4 only, C4/Conc-16 Gy, and C4/Conc-8 Gy × 3 fx (Figure 4a). The single-fraction dose equivalent to the multiple fractions was determined based on a linear–quadratic model [36,37].

C4 at 150 µg/mouse/day was intraperitoneally administered on days 0, 3, and 6 after the tumor volume reached ≥14 mm^3^. One side of the tumor was irradiated using an orthovoltage X-ray irradiator, RF-350 (Rigaku Denki, Tokyo, Japan), under the condition of 180 kV, 15 mA. The rest of the mouse’s body was shielded with more than 4 mm lead blocks. During irradiation, mice were anesthetized with 40 mg/kg/day of Pentobarbital Na diluted in PBS to fix in an in-house jig.

Tumor volume was measured at least every 3 days and calculated with the following formula: (longest diameter)2 × (shortest diameter) × π/6 and binned 3 days for analysis.

### 4.4. In Vitro Irradiation

Cells were irradiated with a Gammacell 40 Exactor (Best Theratronics Ltd., Ottawa, ON, Canada), and irradiation was performed 12–18 h after seeding.

### 4.5. RNA Preparation and Quantitative RT-PCR

RNA samples obtained from cultured LM8 cells 24 h after irradiation according to the protocol of RNeasy Mini kit (QIAGEN, Hilden, Germany) were reverse-transcribed to cDNA as described previously [31]. Quantitative polymerase chain reaction with reverse transcription (qRT-PCR) was performed with *Power*SYBR Green PCR Master Mix (Thermo Fisher Scientific, Waltham, MA, USA). The reactions were run using the ViiA7 Real-Time PCR System (Thermo Fisher Scientific) with an annealing temperature of 60 °C. The gene-specific primer sequence of *Ifnb1* is as follows:

*Ifnb1* Fw  5′-AAGAGTTACACTGCCTTTGCCATC-3′

*Ifnb1* Rv  5′-CACTGTCTGCTGGTGGAGTTCATC-3′

where the GenBank accession number is NM_010510 and the loci of Fw and Rv primers are from the 225th to 248th and from the 336th to 359th of the published base sequences of the mRNA, respectively.

GAPDH was used as the reference gene, with the gene-specific primer sequence of *Ifnb1* as follows:

*Gapdh* Fw 5′-ACCACAGTCCATGCCATCAC-3′

*Gapdh* Rv 5′-CACCACCCTGTTGCTGTAGCC-3′

where the GenBank accession number is NM_001289726 and the loci of Fw and Rv primers are from the 612th to 631st and from the 1041st to 1061st of the published base sequence of the mRNA, respectively.

The 2^−ΔΔCt^ method was used to determine the relative fold expression change of *Ifb1* according to the following formula [60].

ΔΔCt = ΔCt (irradiated sample) − ΔCt (0 Gy sample)

where ΔCt is calculated using the following formula.

ΔCt = Ct (*Ifnb1*) − Ct (*Gapdh*).

### 4.6. Flow Cytometry

To analyze the tumor immune microenvironment after irradiation, mice untreated or treated by a single fraction of 10 Gy, 16 Gy, or 8 Gy × 3 fx were sacrificed 3 days after the last delivery of irradiation. A single cell suspension was prepared as previously described [31,56,61]. Briefly, harvested tissue was dissociated with Collagenase IV (Sigma Aldrich, Tokyo, Japan) and DNAse II (Sigma Aldrich). After blocking Fc receptors with anti-CD16/32 antibody (clone nr. 93, BioLegend, San Diego, CA, USA), cells were incubated with anti-CD4-APC antibody (RM4-5, eBioscience) on ice for 30 min at a ratio of 1:80 with FACS buffer (PBS containing 10% FBS and 0.5 mM EDTA). Then, cells were fixed, permeabilized, and incubated with anti-Foxp3-PE antibody (FJK-16s, eBioscience) on ice for 30 min at a ratio of 1:80. Fixation and permeabilization were conducted using a Foxp3 staining buffer kit (eBioscience). After washing twice with permeabilization buffer followed by washing once with FACS buffer, cells were analyzed with a FACS verse flow cytometer (Beckton Dickinson, Franklin Lakes, NJ, USA). Data were analyzed using Flow Jo ver. 10 (Tommy Digital Biology, Tokyo, Japan).

### 4.7. Quantification of Cytosolic Double-Stranded DNA

Irradiated cells in the cytoplasm were isolated using NE-PER^TM^ Nuclear and Cytoplasmic Extraction Reagents (Thermo Fisher Scientific). Double-stranded DNA (dsDNA) in the cytoplasm of 1 × 10^6^ live LM8 cells was quantified with the SpectraMax^®^ Quant^TM^ AccuClear Nano dsDNA Assay Explorer kit (Molecular devices, San Jose, CA, USA) 24 h after the last delivery of irradiation. The samples were analyzed by Varioskan (Thermo Fisher Scientific, Waltham, MA, USA).

### 4.8. Measurement of IFN-β Secretion

Cell-free supernatants were collected 24 or 48 h after the last irradiation. A mouse IFN-β high-sensitivity ELISA kit (PBL Assay Science, Piscataway, NJ, USA) was used to quantify secreted IFN-β. The sensitivity is 0.94 pg/mL according to the manufacturer’s specification. All procedures were performed according to the manufacturer’s manual, and the concentration measurement was performed with Varioskan (Thermo Fisher Scientific). The concentration [pg/mL] per 10^5^ live cells was analyzed according to a previous study [35]. 

### 4.9. Western Blotting

LM8 cells were lysed in PierceTM RIPA buffer (Thermo Fisher Scientific) supplemented with 1% 100× Protease/Phosphatase inhibitor (Thermo Fisher Scientific) 24 h after the last irradiation. Whole cell extracts were centrifuged at 14,000 rpm for 15 min at 4 °C. The protein concentration of each sample was determined with a Pierce^TM^ BCA Protein Assay Kit (Thermo Fisher Scientific) by measuring the absorption using Varioskan (Thermo Fisher Scientific) according to the manufacturer’s instructions. For SDS-PAGE, samples were run using 10% unstained precast gel (Bio-Rad, Hercules, CA, USA) under the condition of 70 kV for 10 min followed by at 100 kV for 1 h. The wet tank method was used to transfer proteins to a PVDF membrane under the condition of 100 kV for 1 h. The membrane was blocked with blocking buffer (TBS-T containing 5% BSA or non-fat milk powder) for 1 h at room temperature before reacting with cGAS (clone nr. D3080, Cell Signaling Technologies, Danvers, MA, USA), STING (D1V5L, Cell Signaling Technologies), IRF3 (D83B9, Cell Signaling Technologies), and phospho-IRF3 (S396) (D601M, Cell Signaling Technologies) specific primary antibodies overnight at 4 °C at a ratio of 1:1250 with blocking buffer. The membrane was incubated with horseradish peroxidase-conjugated secondary antibody (Cell Signaling Technologies) for 1 h at room temperature at a ratio of 1:3000 with blocking buffer. SuperSignal^TM^ West Pico PLUS Chemiluminescent Substrate (Thermo Fisher Scientific) was used for chemiluminescence, and specific bands were visualized utilizing the ChemiDoc + XRS or ChemiDoC Touch imaging systems (Bio-Rad, Hercules, CA, USA). Densitometric quantification of the Western blot signals was performed using ImageJ (National Institutes of Health, Bethesda, MD, USA), an open source program for image processing. The region of interest was set on the bands of the specific protein, followed by a measurement of the signal density in the area of each blot. Each calculated value in the specific sample was normalized by the corresponding value of the loading control (β-actin).

### 4.10. Statistics

For the tumor volume comparison, the Mann–Whitney U test or Steel test were performed for a one-on-one comparison or multiple comparisons, respectively. Overall survival was analyzed by the Kaplan–Meier method, and statistical significance was calculated by a log-rank test. Tumor volume graphs are presented as mean ± SEM (standard error of the mean).

The chi-squared test was used for the comparison of the responders and the acute skin reaction between the high- and intermediate-dose regimens. 

Dunnet’s multiple comparison test was conducted for the comparison of the flow cytometry results between the 0, 10, 16 Gy, and 8 Gy × 3 fx groups. Tukey–Kramer’s honestly significant difference test was used to compare the concentration of IFN-β in the supernatant between 10 Gy, 16 Gy, and 8 Gy × 3fx 48 h after irradiation, and dsDNA in cytoplasm and fold expression change of the IFN-b gene between the 0 Gy, 10 Gy, 16 Gy, and 8 Gy × 3 fx groups. A two-tailed Student’s t-test was used to compare the IFN-β concentration between 8 Gy × 3 fx 24 h after irradiation and 8 Gy × 3 fx 48 h after irradiation. The graphs of flow cytometry and all in vitro experiments are presented as mean ± standard deviation.

## 5. Conclusions

Our data demonstrated that high-dose irradiation strongly activated the type-I IFN pathway and induced IFN-β secretion. Radiation dose escalation that almost eradicates tumors was crucial in C4 therapy to enhance the antitumor response in both local and distant tumors, as well as for prolonged overall survival regardless of fractionation in osteosarcoma. These data provide rationale for the development of new therapeutic strategies for patients with osteosarcoma. Further dose escalation by hypofractionated or a single fraction of radiation delivery may provide greater improvement of the local and distant tumor controls. However, even the use of high-precision radiation therapy techniques using X-ray limits dose escalation because of possible normal tissue toxicities. Owing to the superior physical and biological characteristics of carbon ion beams, dose escalation using carbon ion beams with C4 may provide a better antitumor immune response than photon beams. Further studies using carbon ion beams with immune checkpoint blockade are necessary to achieve much better control of local and distant tumors for osteosarcoma.

## Supplementary Materials

The following are available online at https://www.mdpi.com/2072-6694/12/6/1546/s1, Figure S1: Pie charts showing the ratio between complete and partial response (CR + PR) and stable and progressive disease (SD+PD) of the abscopal tumor between C4 only and C4 with intermediate dose (10 Gy, 4.5 Gy × 3 fx and 2 Gy × 8 fx) groups, Figure S2: Concentration of IFN-β in the cell culture supernatant 24 and 48 h after the final delivery of irradiation at 0 Gy, 2 Gy, and 30 Gy, Figure S3: Untrimed Western blot data. Figure S4: Comparison of tumor volumes between sequential and concurrent combinations of high-dose hypofractionated irradiation with C4 therapy.
