# Supplementary material for: Radiation Dose Escalation Is Crucial in Anti-CTLA-4 Antibody Therapy to Enhance Local and Distant Antitumor Effect in Murine Osteosarcoma
Source: Cancers (Basel). 2020 Jun 12;12(6):1546. doi: 10.3390/cancers12061546 (PMC7352693; doi:10.3390/cancers12061546)
Supplement: Supplementary file 1 [file cancers-12-01546-s001.pdf]

cancers

Article

Radiation Dose Escalation is Crucial in Anti-CTLA-4 Antibody Therapy to Enhance Local and Distant Antitumor Effect in Murine Osteosarcoma

Wataru Takenaka, Yutaka Takahashi, Keisuke Tamari, Kazumasa Minami, Shohei Katsuki, Yuji Seo, Fumiaki Isohashi, Masahiko Koizumi and Kazuhiko Ogawa

Supplementary Materials:

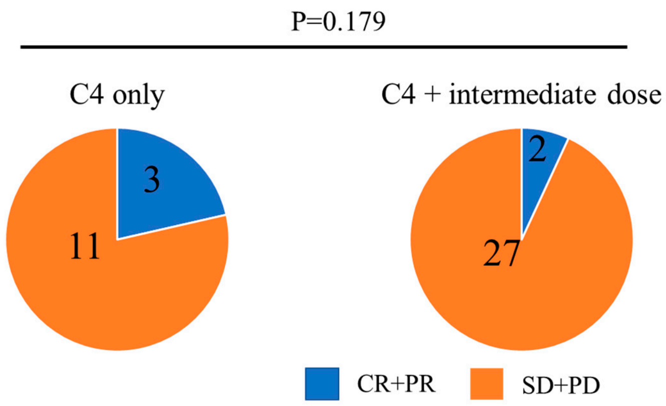

Figure S1. Pie charts showing the ratio between complete and partial response (CR+PR) and stable and progressive disease (SD+PD) of the abscopal tumor between C4 only and C4 with intermediate dose (10 Gy, 4.5 Gy × 3 fx and 2 Gy × 8 fx) groups.

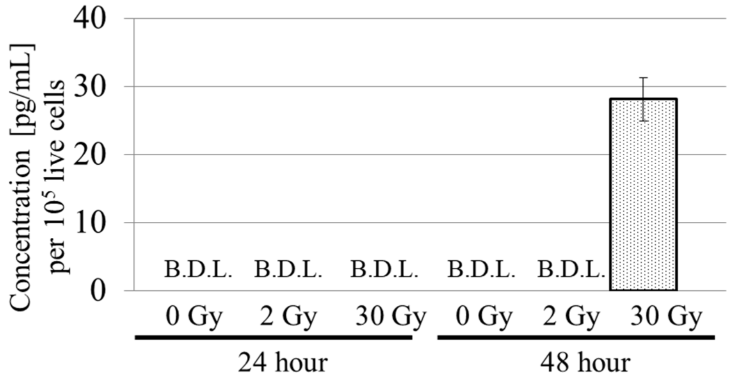

Figure S2. Concentration of IFN-β in the cell culture supernatant 24 and 48 h after the final delivery of irradiation at 0 Gy, 2 Gy, and 30 Gy. Error bars represent SD. Abbreviation; B.D.L.: Below detection limit.

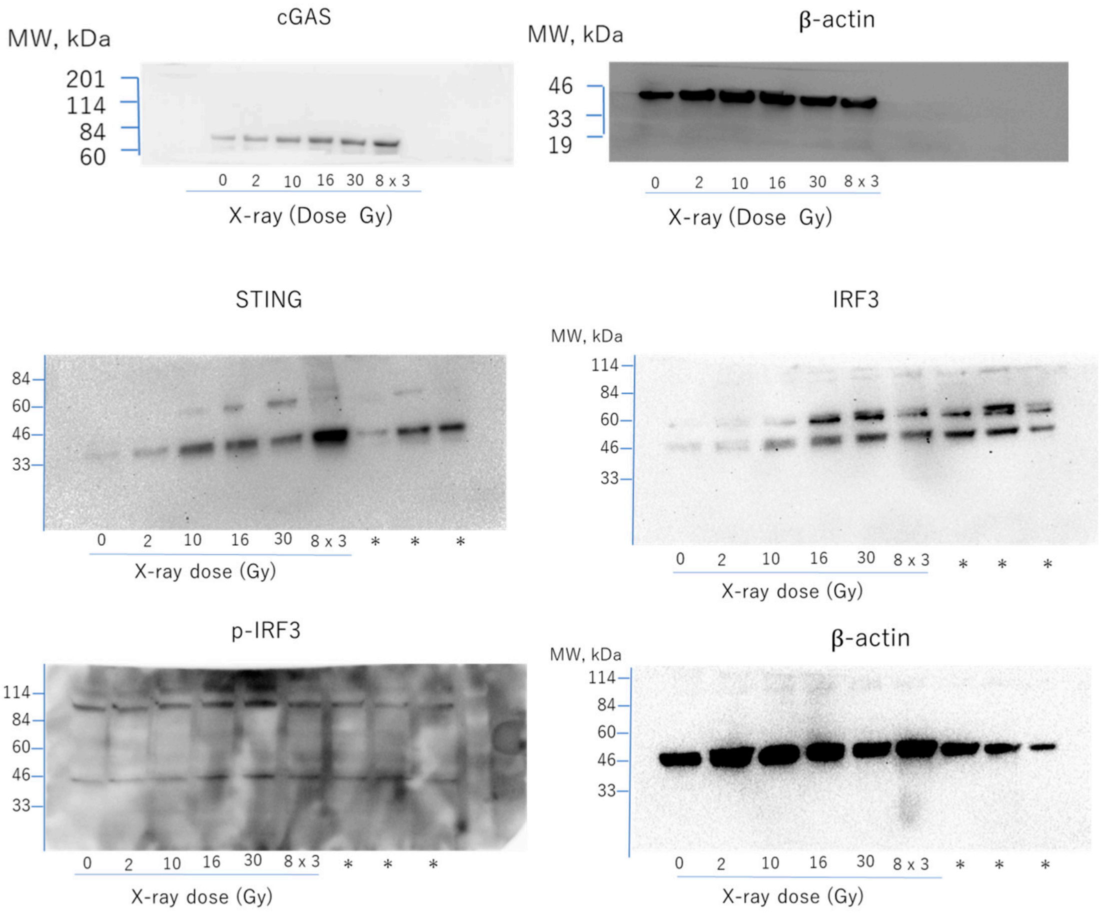

Figure S3. Untrimmed pictures of the western blot in Figure 3 (b). *; Unrelated samples to this study.

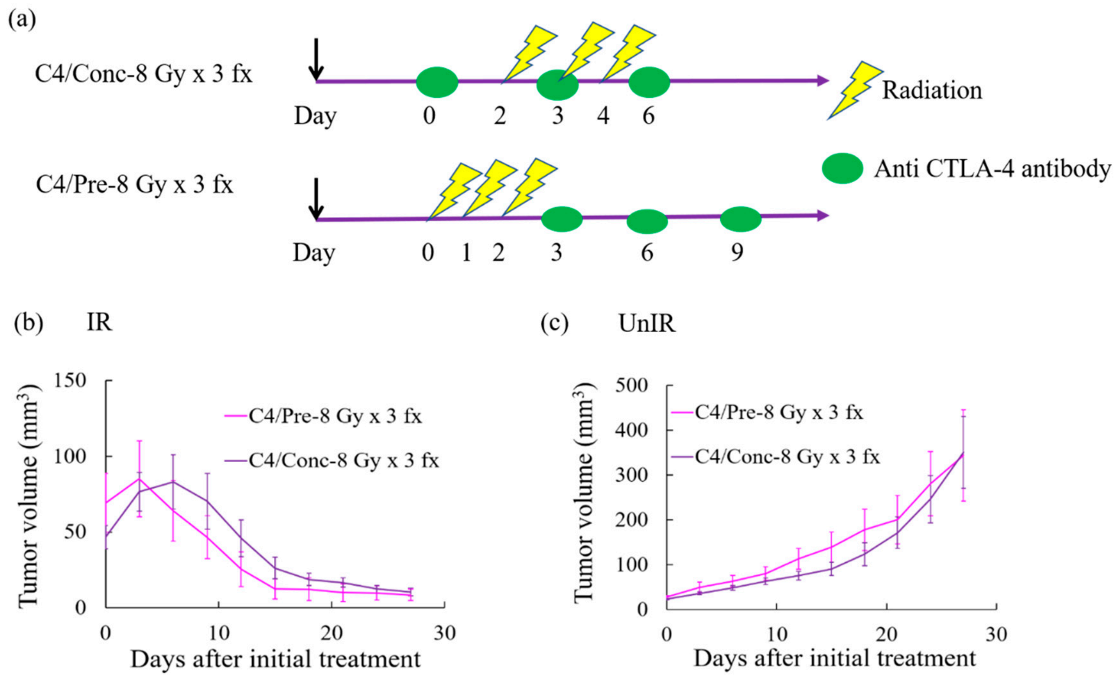

Figure S4. Comparison of tumor volumes between sequential and concurrent combinations of high-dose hypofractionated irradiation with C4 therapy. (**a**) Schematic diagram of treatment regimens. (**b**) Tumor volume change after initial treatment of the irradiated tumor. (**c**) Tumor volume change after initial treatment of the unirradiated tumor. Error bars represent SEM.

© **2020** by the authors. Licensee MDPI, Basel, Switzerland. This article is an open access article distributed under the terms and conditions of the Creative Commons Attribution (CC BY) license (http://creativecommons.org/licenses/by/4.0/).
